# Sponge Gold

**Published:** 1854-04

**Authors:** A. A. Blandy


### ARTICLE X.

### *Sponge Gold.* By A. A. BLANDY, M. D., D. D. S.

THE use of other preparations of gold than foil for filling teeth, has, within the last three or four years, attracted considerable attention, and to Dr. Watts, chemist, of Utica. N. Y., the members of the dental profession are indebted for an article of crystalline sponge that bids fair to come into general use. This gentleman placed in my hands, a few months since, some specimens which he had prepared, with a request that I should submit them to such tests as I might deem necessary to satisfy myself fully with regard to their usefulness and value as compared with other preparations of the kind that had been made for the same purpose. Having charge of the large Infirmary of the Baltimore Dental College, where five operating chairs are constantly occupied, I at once instituted a series of experiments with this preparation, the result of which, I now take the liberty of making public. That the experiments might be fair and thorough, the use of foil, was, for the time, laid wholly aside, and the crystalline sponge substituted in all cases, no matter what tooth or the locality of the cavity in it to be filled. At first considerable difficulty was experienced in the working and management of the material, our instruments being sharp or wedge-pointed carriers, adapted only for the introduction of foil, whereas for the introduction of the crystalline sponge, blunt concave-pointed instruments, roughened on the surface, are, for the most part, better suited for introducing and consolidating the metal. If the crystals are separated from the spongy mass before they are brought firmly in contact with each other, they escape into the mouth and are lost, and to prevent which, when the instruments commonly employed are used, requires a great deal of tact and skillful management on the part of the operator. But this inconvenience and loss, decreased day by day, as the operators acquired experience and familiarity in the use of the article, until finally beautiful fillings were made with it even in the approximal surfaces of front teeth, but I am compelled to say, that in the latter class of fillings, such result was only obtained at the expense of a greater amount of time and labor than is required with foil, and also with considerable more waste of material.

When the cavity in the tooth is deep, great care is necessary to prevent choking the orifice, as the crystals unite as soon as they are brought firmly in contact with each other. When this happens it is with great difficulty that an instrument can be forced through the incrustation. The process of consolidating the gold, therefore, should be commenced at the bottom, and against the walls of the cavity. Unless this is done, it will be impossible to give to every part of the filling absolute solidity. Again, if a sufficient quantity of the sponge to fill the cavity is not introduced before the mass is completely consolidated, it is almost impossible to add more, if the surface of that which was first introduced has become covered with moisture, unless the walls of the remaining portion of the cavity furnish sufficient mechanical support for its retention, or the gold already introduced presents a surface with one or more deep depressions capable of holding the fresh supply. This, of course, need not occur in filling a cavity in the grinding surface of a tooth, but in the approximal it is particularly liable to happen. For filling a cavity in the grinding surface of an inferior molar or bicuspid, this preparation of gold is superior to any foil I have ever used.

No one need expect in his first attempts to use it, to be able to do so to his entire satisfaction. Nearly as much tact and experience are required for its skillful management as is needed to make a good filling of foil.

The cohesive properties of the crystalline sponge as prepared by Dr. Watts is vastly greater than foil, as the crystals when brought in absolute contact with each other, if there be no interposition of moisture, unite so firmly as to render subsequent separation utterly impossible, but this property becomes less and less manifest in the application of fresh sponge, in proportion to the solidity which has been imparted to the crystals by the pressure which has been applied to them. It is evident, then, in the introduction of the filling. the portion last applied should be a little more than sufficient to fill the cavity, as any after application may prove exceedingly troublesome, unless the gold has been kept perfectly dry and the instruments employed in condensing are of such construction as to leave a rough uneven surface which will render adhesion of the superadded gold more complete.

I was led to believe at first, that the crystallized gold, from its extraordinary adhesive property, might be used advantageously in connection with foil, but I regret to say that my hopes, as yet, have not been realized. I thought it possible that when a sufficient quantity of foil had not been used to fill the cavity completely, the sponge might be added and welded to it until the filling should be made flush with the surface of the tooth, and that in this way the latter might prove an invaluable adjunct to the former, but the experiments which I have thus far made have failed to confirm the suggestive hope which had thus occurred to my mind, they having proved wholly abortive. It cannot be made to adhere to an old filling, or, if so, more experience is required to make it do so than I have had in the use of the article as yet.

Among the inconveniences which I have experienced in using it, I will mention one more, and it is this: It being introduced in small lumps or irregular masses, is apt to condense unequally, leaving little depressions on the surface, alike difficult to erase, as to remedy by addition. If, when the protruding portion of a filling, made in the ordinary way with foil, is filed down to a level with the surface of the tooth, a minute depression is found where the gold has not been thoroughly consolidated, a perforation may be made in it with a strong sharp or wedge-pointed instrument which may be filled and the entire surface rendered perfectly smooth and susceptible of a high polish. But this cannot be done with the crystalline sponge, nor can the imperfection be so easily remedied. It will offer more resistance to the pressure of the instrument, and if a depression is made, it will be of a saucer shape, and of course more difficult to fill and finish than a perforation made in a filling of foil under like circumstances. The only certain remedy is to *drill* a hole into the defective part and fill this in the same manner as if it were the original cavity.

For these reasons it is desirable that the cavity should always be more than full when the gold is thoroughly consolidated, and in this case, a filling of unsurpassed beauty of finish can be made with it.

A more extended experience than any one has yet had with crystallized gold is necessary to an absolutely correct judgment with regard to its relative value as compared with foil, but from the experiments I have made, I am disposed to believe it will prove an invaluable acquisition to dental practice. Indeed, I almost feel warranted in the assertion that with an equal amount of labor better fillings can be made with it in the grinding surfaces of the molar and bicuspid teeth, and in large cavities easy of access in the approximal surfaces of most teeth, than with foil. But in small cavities, and especially in the approximal surfaces of teeth, not easily reached, and when partially covered by the edge of the gum, foil can certainly be used with much greater facility and certainty.

I have seen some most beautiful fillings in almost every locality in the teeth, and even plate of firm texture made from this preparation of gold; also, a massive and well finished gold ring, and although we do not think it will ever supersede the use of Abbey's and Morgan's foil, we would urge for it a fair trial. That its use, to a considerable extent at least, will become general amongst dentists, we scarcely entertain a doubt.

Since the foregoing was written, an article on the use of this preparation of gold, which will be found in another part of the present number of the Journal, has been received from Dr. W. H. Dwinelle, who, as will be seen, has submitted it to the severest trials, and with the most satisfactory and gratifying results.

